# Association between Dietary Intake of Flavonoids and Cancer Recurrence among Breast Cancer Survivors

**DOI:** 10.3390/nu13093049

**Published:** 2021-08-30

**Authors:** Minjung Cheon, Minsung Chung, Yongsoon Park

**Affiliations:** 1 Department of Food and Nutrition, Hanyang University, 222 Wangsimni-ro, Seongdong-gu, Seoul 04763, Korea; min10jung04@hanyang.ac.kr; 2 Department of Surgery, Hanyang University Hospital, 222 Wangsimni-ro, Seongdong-gu, Seoul 04763, Korea

**Keywords:** breast cancer, flavonoids, flavonoid-rich foods, obesity, recurrence, survival

## Abstract

Intake of flavonoids is associated with the incidence of breast cancer, but the association between the intake of flavonoids and cancer recurrence is unclear. This study aimed to investigate the hypothesis that intake of flavonoids and flavonoid-rich foods is negatively associated with cancer recurrence. Among 572 women who underwent breast cancer surgery, 66 patients had a cancer recurrence. Dietary data were collected using a structured 24-h dietary recall, and intake of flavonoids was calculated based on the Korea Rural Development Administration flavonoid database. Among overweight and obese patients, disease-free survival was associated with intake of flavonoids (*p* = 0.004) and flavonoid-rich foods (*p* = 0.003). Intake of flavonoids (hazard ratio (HR) = 0.249, 95% confidence interval (CI): 0.09–0.64) and flavonoid-rich foods (HR = 0.244, 95% CI: 0.09–0.66) was negatively associated with cancer recurrence after adjusting for confounding factors in overweight and obese patients. Consumption of flavonoids and flavonoid-rich foods was lower in overweight and obese patients with cancer recurrence than those without recurrence and in normal-weight patients. This study suggests that intake of flavonoids and flavonoid-rich foods could have beneficial effects on cancer recurrence in overweight and obese breast cancer survivors.

## 1. Introduction

Breast cancer is the most common cancer and the leading cause of cancer-related deaths among women worldwide [1]. Breast cancer recurrence leads to an increased risk of metastases and decreased survival [1]. Diet is a major modifiable risk factor for breast cancer incidence, recurrence, and mortality [2]. Our previous study showed that anti-inflammatory diets were related with cancer recurrence and overall mortality in breast cancer patients [3]. A meta-analysis of epidemiological studies reported that vegetable intake was negatively associated with cancer-related mortality [4] and breast cancer incidence [5]. Vegetables are the main source of flavonoids, including flavonols and flavones, which are natural antioxidants that reduce oxidative DNA damage [6].

Epidemiological studies have suggested that dietary intake of flavonols and flavones had a beneficial effect on the incidence of breast cancer [7,8,9,10]. Breast-cancer-specific mortality was also lower in those with the highest quintile of flavone intake than those with the lowest quintile of flavone intake [11]. However, Kyrø et al. [12] showed no association between intake of flavonoids and breast-cancer-related mortality in patients with breast cancer. This inconsistency could partly be due to the effect of interaction between flavonoids and fat mass on the risk of breast cancer since intake of flavonoids was associated with weight loss in the Nurses’ Health Study [13] and lower body mass index (BMI) in a study using the National Health and Nutrition Examination Survey (NHANES) [14]. Bosetti et al. [7] observed that dietary intake of flavones was associated with incidence of breast cancer in participants with a BMI of ≥25 kg/m^2^, but not in those with a BMI of <25 kg/m^2^, suggesting that intake of flavonoids could be more beneficial for obese breast cancer patients. Flavonoids have been shown to inhibit body fat accumulation by reducing the activity of fatty acid synthase (FAS) in obese mice fed a high-fat diet [15].

There has been no study evaluating the association between dietary intake of flavonoids and breast cancer recurrence. Therefore, this study aimed to investigate the hypothesis that dietary intake of flavonoids and flavonoid-rich foods is associated with cancer recurrence in patients with breast cancer. In addition, the association between flavonoid intake and cancer recurrence was compared between normal-weight and overweight/obese patients with breast cancer.

## 2. Materials and Methods

### 2.1. Patients

**Figure 1:**
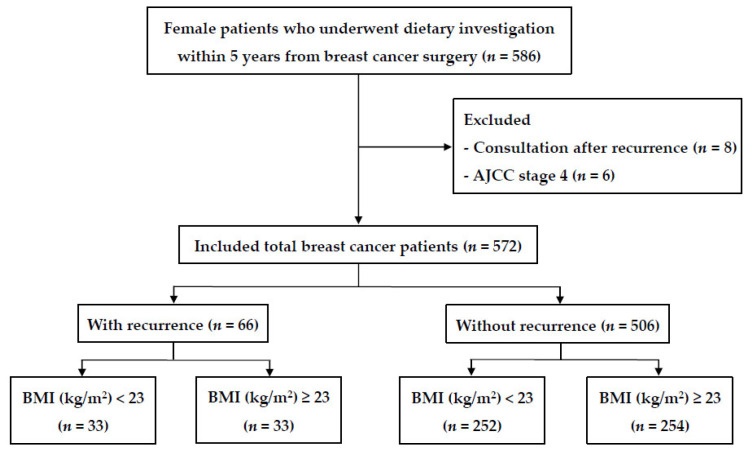
Flowchart of the study patient selection process. AJCC, American Joint Committee on Cancer; BMI, body mass index.

The study patients were recruited from a currently ongoing series of dietary survey projects on breast cancer. A total of 586 female patients who underwent breast cancer surgery at the breast cancer clinic of Hanyang University Seoul Hospital from March 2011 to April 2020 and who completed the dietary survey within five years from the date of breast cancer surgery were enrolled (Figure 1). Patients were excluded if they completed the dietary survey after cancer recurrence (*n* = 8) or if they had stage IV breast cancer (*n* = 6). After exclusion, 572 patients participated in the study.

Patients were followed up from the date of surgery until the time of the first confirmed cancer recurrence, death, or the end of September 2020, whichever occurred first. Patients who were lost to follow-up were censored at the date of the last visit to the hospital. Recurrence included local or regional cancer recurrence, development of contralateral breast cancer, and distant metastasis. Local recurrence was defined as recurrence in the residual breast tissue or breast wall, and regional recurrence was defined as recurrence in the ipsilateral regional lymph nodes.

This study was conducted in accordance with the Declaration of Helsinki, and all procedures were approved by the Institutional Review Board of Hanyang University Hospital (HYU 2010-02-001-031) and Hanyang University (HYI-15-070). Before enrolment, written informed consent was obtained from all patients.

### 2.2. Data Collection

Data on age, height, body weight, estrogen receptor (ER) status, progesterone receptor (PR) status, human epidermal growth factor receptor 2 (HER2) status, histologic grade, tumor size, lymph node metastasis status, and type of treatment were obtained from the medical records and histopathology reports. Tumor and lymph node stages were calculated according to the seventh edition of the American Joint Committee on Cancer (AJCC) staging system [16]. Overweight, obesity, and abdominal obesity were defined according to the Korean Society for the Study of Obesity [17].

Biochemical analyses were performed using blood samples collected from patients under fasting within a 2-month interval from the dietary survey. Serum levels of total protein, albumin, alkaline phosphatase, aspartate aminotransferase, alanine aminotransferase, white blood cell, hemoglobin, hematocrit, triglycerides, high-density lipoprotein cholesterol, low-density lipoprotein cholesterol, cholesterol, and cancer antigen 15-3 were obtained from the medical charts.

### 2.3. Intake of Dietary Flavonoids

A trained dietitian collected dietary data using a structured 24-h recall after breast cancer surgery, and dietary intake was analyzed using CAN-pro web, version 5.0 (computer-aided nutritional analysis program; Korean Nutrition Society, Seoul, Korea). Dietary intake of flavonols and flavones was estimated by matching 270 food items in the Korea Rural Development Administration flavonoid database [18]. Intake of flavonols and flavones was calculated by multiplying the content (mg) of flavonols and flavones by the amount of food (g) consumed by patients. Among the 165 foods consumed by our patients, foods rich in flavonols and flavones were defined as foods with a content of flavonols and flavones higher than the mean content of flavonols (>38.06 mg/100 g) or flavones (>62.01 mg/100 g) [19]. Flavonol-rich foods included green tea, castor aralia, red lettuce, butterbur, kale, mustard, onion, and seasoned cabbage, and flavone-rich foods included pepper leaf, mung bean, shepherd’s purse, Chinese cabbage, green pepper, red pepper, red lettuce, and celery.

### 2.4. Statistical Analyses

The Kolmogorov–Smirnov test was used to assess the normal distribution of the variables. Continuous variables are presented as mean ± standard deviation (SD). Differences between patients with and without cancer recurrence were verified using a one-way analysis of variance (ANOVA) or the Kruskal–Wallis test, followed by the least significant difference (LSD) post hoc test. Proportions of nominal variables, estimated using the chi-square test, are presented as the number of patients and percentage distribution. In the multivariate models, covariates with a *p*-value of < 0.20 were selected as confounding factors and included in the fully adjusted model [20]. Age, waist circumference, alcohol drinking, tumor size, lymph node metastasis, histological grade, and energy intake were identified as confounding factors. Biochemical evaluation of the blood samples was performed using a ranked one-way analysis of covariance (ANCOVA) after adjusting for confounding factors. The interactions between flavonoid intake and BMI were tested using a two-way ANOVA. Intake of flavonoids and flavonoid-rich foods was categorized into tertiles based on the distribution of patients, and the lowest tertiles of flavonoid and flavonoid-rich food intake were used in the reference group. The hazard ratios (HRs) and 95% confidence intervals (CIs) were calculated using multivariable Cox proportional hazards regression analysis after adjusting for confounding factors to evaluate the association between dietary intake of flavonoids/flavonoid-rich foods and breast cancer recurrence. The Kaplan–Meier method was used to calculate cumulative survival probabilities, and the difference between the survival curves was assessed using the log-rank test. All data analyses were performed using SPSS 25.0 (SPSS Inc., Chicago, IL, USA), and a *p*-value of <0.05 was considered statistically significant.

## 3. Results

### 3.1. Characteristics of Patients with Breast Cancer

Overweight and obese patients with cancer recurrence had a higher BMI and waist circumference, a larger tumor size, and more lymph node metastases than overweight and obese patients without cancer recurrence and normal-weight patients (Table 1). Patients with cancer recurrence were more likely to be current drinkers than those without cancer recurrence. Moreover, overweight and obese patients without cancer recurrence were older than other patients. There were no significant differences in family history of breast cancer, smoking status, AJCC stage, type of treatment, histological grade, ER status, hormone receptor status, HER2 amplification status, and subtype between the groups (Table 1). All biochemical measurements were not significantly different between the patients (Table S1).

### 3.2. Association between Intake of Flavonoids/Flavonoid-Rich Foods and Cancer Recurrence

Overweight and obese patients with cancer recurrence had a lower intake of flavonoids, flavonols such as quercetin and kaempferol, and flavones such as apigenin and luteolin than other patients after adjusting for confounding factors (Table 2). Intake of flavonoid-rich foods, including flavonol-rich and flavone-rich foods, was consistently lower in overweight and obese patients with cancer recurrence than in other patients after adjusting for confounding factors (Table 2). However, there was no difference in intake of isorhamnetin between the patients. Additionally, there was a significant interaction between intake of flavonoids and flavonoid-rich food and BMI status on cancer recurrence, except for isorhamnetin intake. There was no significant association between intake of flavonoids and flavonoid-rich foods and cancer recurrence in all patients (Tables S2 and S3). However, among overweight and obese patients, intake of flavonoids, flavonols, and flavones, except isorhamnetin, was negatively associated with risk of cancer recurrence before and after adjusting for confounding factors (Table 3). The same association was consistently observed for flavonoid-rich foods, flavonol-rich foods, and flavone-rich foods (Table 4). Additionally, there was a negative association between risk of cancer recurrence and intake of flavonoids/flavonoid-rich foods as a continuous variable (Table 3; Table 4).

### 3.3. Association between Intake of Flavonoids/Flavonoid-Rich Foods and Disease-Free Survival

**Figure 2:**
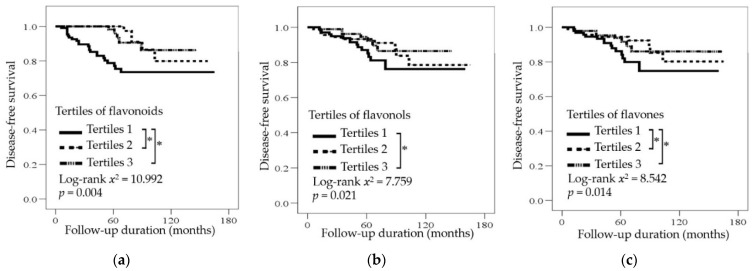

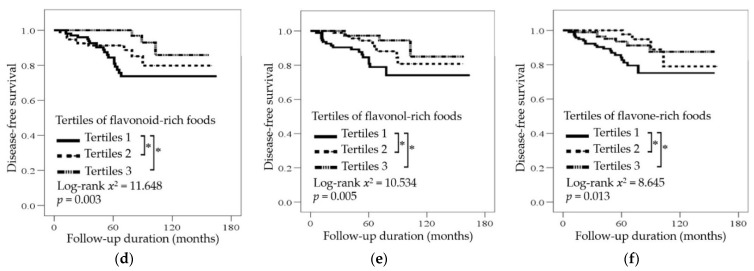
Cumulative disease-free survival according to the tertiles of flavonoid (**a**–**c**) or flavonoid-rich food intake (**d**–**f**) in overweight and obese patients with breast cancer. Statistical significance was determined using the log-rank test and the Kaplan–Meier method; * *p* < 0.05.

Among overweight and obese patients with breast cancer, the median period of disease-free survival and overall survival was 43 months (range, 6–103 months; Figure 2a–f) and 72 months (range, 12–120 months), respectively (Figure S1a,b). Intake of flavonoids and flavonoid-rich foods was not significantly associated with disease-free survival and overall survival in all patients according to the Kaplan–Meier survival curves (Figure S2a–d). However, there was a significant correlation between dietary flavonoids, such as flavonols and flavones, and flavonoid-rich foods, such as flavonol-rich and flavone-rich foods, and disease-free survival in overweight and obese patients (Figure 2). Patients in the middle and highest tertiles of dietary flavonoids and flavone- and flavonoid-rich foods had longer survival than those in the lowest tertile (Figure 2a,c–f). Patients in the highest tertile of flavonol intake had prolonged survival compared to those in the lowest tertile (Figure 2b). Furthermore, intake of flavonoids and flavonoid-rich foods was not significantly associated with overall survival in overweight and obese patients (Figure S1a,b).

## 4. Discussion

This is the first study to show that dietary intake of flavonoids and flavonoid-rich foods was negatively associated with cancer recurrence among overweight and obese patients who underwent breast cancer surgery. No previous study has shown an association between dietary intake of flavonoids and the risk of breast cancer recurrence. However, a meta-analysis of epidemiological studies reported that intake of flavonols and flavones was negatively associated with the risk of breast cancer incidence [9]. Intake of flavonoid-rich foods, such as cauliflower, winter squash, spinach, carrots, pepper, garlic, onion, and beans, was also negatively associated with the incidence of breast cancer [21,22,23,24,25]. In addition, cancer recurrence was negatively associated with intake of vegetables [26], flavonoid-rich soy foods [27], and green tea [28] in breast cancer patients. In human breast cancer cells and xenograft animal models, flavonoids such as quercetin, kaempferol, apigenin, and luteolin have been shown to exert anti-cancer effects by inhibiting cell growth through apoptosis [29].

In previous epidemiological studies, the risk of breast cancer incidence was lower in participants with a higher than average intake of flavonoids in the United States [30] and in China [8]. The average intake of flavonoids is 23.0 mg/day in obese Korean women according to the Korea National Health and Nutrition Examination Survey (KNHANES) [31]. The present study showed that the risk of cancer recurrence was significantly lower in obese breast cancer patients with a higher than average intake of flavonoids. In addition, Feng et al. [32] showed that serum concentration of flavonoids was negatively associated with risk of breast cancer incidence. Jodynis-Liebert et al. [33] reported that high concentrations of flavonoids reduced tumor cell growth in an in vitro study. These results suggest that individuals should consume an amount of flavonoids higher than the average value to reduce not only the risk of incidence but also the recurrence of breast cancer.

**Scheme 1:**
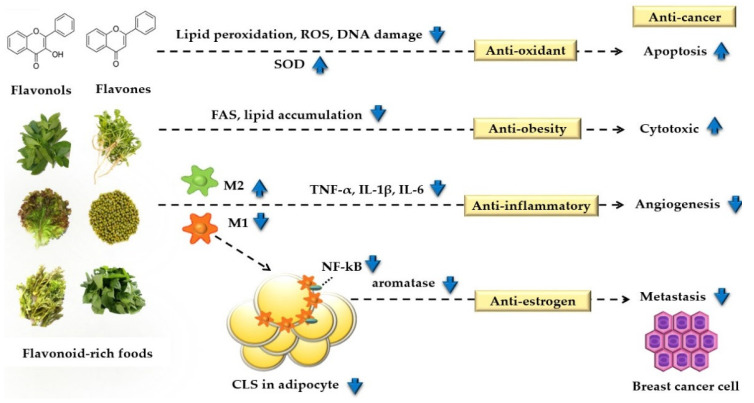
Schematic diagram of the antioxidant, anti-obesity, anti-inflammatory, and anti-estrogen pathways regarding the effect of flavonoids on obesity-induced breast cancer. SOD, superoxide dismutase; ROS, reactive oxygen species; FAS, fatty acid synthase; NF-κB, nuclear factor-κB; TNF-α, tumor necrosis factor-alpha; IL-6, interleukin-6; IL-1β, interleukin-1β; CLS, crown-like structure.

In the present study, a significant interaction was observed between flavonoids and BMI status, suggesting that the effect of flavonoid intake on cancer recurrence might be different according to the BMI status of breast cancer patients. Previous studies observed that intake of flavonoids was negatively associated with the incidence of breast cancer in participants with BMI ≥ 25 kg/m^2^, but not in those with BMI < 25 kg/m^2^ [7]. In addition, a randomized controlled trial showed that supplementation of both flavonoid-rich green tea beverage and extract decreased body weight and BMI only in obese subjects [34]. BMI was correlated with plasma lipid peroxidation [35] and FAS expression [36] in obese patients but not in normal-weight patients. Lipid peroxidation [37] and FAS expression [38] were correlated with breast cancer recurrence in patients with breast cancer. Furthermore, flavonoids, as an antioxidant, increased apoptosis of cancer cells by reducing lipid peroxidation, reactive oxygen species, and DNA damage and by increasing superoxide dismutase [39] (Scheme 1). Flavonoids also inhibited lipid accumulation and FAS in breast cancer cells, suggesting that these anti-obesity effects of flavonoids increased the cytotoxicity of cancer cells [40]. Additionally, flavonoids’ immune regulation function is related to conversion of the adipocyte macrophage phenotype M1 (pro-inflammatory) to M2 (anti-inflammatory) [41]. Thus, the anti-inflammatory effect of flavonoids inhibits angiogenesis of cancer cells by decreasing inflammatory cytokines such as tumor necrosis factor-alpha, interleukin (IL)-1β, and IL-6 [42], and the anti-estrogen effect prevents metastasis of cancer cells by decreasing aromatase activity [43]. These studies support our results showing that intake of flavonoids can prevent cancer recurrence in overweight and obese patients.

In the present study, breast cancer recurrence was associated with intake of flavonols such as quercetin and kaempferol, but not with intake of isorhamnetin. Our patients consumed 78%, 15%, and 7% dietary flavonols in the form of quercetin, kaempferol, and isorhamnetin, respectively. Consistent with this, the KNHANES reported that Korean adults consumed 76%, 20%, and 4% dietary flavonols in the form of quercetin, kaempferol, and isorhamnetin, respectively. These results indicate that the effect of isorhamnetin on breast cancer might be weak because isorhamnetin contributes to the smallest proportion of flavonols [44]. Among the flavonols, the inhibitory effect on cell proliferation, cytotoxic effects on cancer cells, and antioxidative effects of isorhamnetin were weaker than those of quercetin and kaempferol in human breast carcinoma cells [45,46].

Dietary intake of flavonoids [47] and vegetables [48] was negatively associated with all cancer-specific mortality. The survival of nude mice inoculated with breast cancer cells was also prolonged with increasing doses of flavonoids when administered orally [49]. In addition, mortality was lower in patients with breast cancer in the highest quintile of flavone intake than in those in the lowest quintile [11]. However, in patients with breast cancer, dietary intake of flavonoids and flavonoid-rich foods was not associated with mortality [11,12]. A meta-analysis of cohort studies and randomized controlled trials also showed that intake of vegetables was not associated with mortality and overall survival in patients with breast cancer [50]. Consistent with the previous studies, the present study showed no association between survival and flavonoid intake, but among obese patients, survival was associated with intake of flavonoids and flavonoid-rich foods.

The present study has a few limitations. First, the quantities of flavonoids in foods might differ according to the species, growth condition, and maturation, preparation, and food-processing methods [51]. These factors, which were not accounted for in the nutrient database, could contribute to random errors in the intake assessment and result in bias toward the null. In addition, flavonoid intakes were calculated based on the database, which had limited food coverage, and thus might be underestimated. Second, dietary intake was measured once through a 24-h recall, which might not be sufficient to determine the patient’s usual intake. Third, although adjustments were made for confounders, unmeasured factors such as the severity of comorbid diseases, lifelong eating behavior, and social factors including income and jobs could have affected the results of this study. Lastly, Ambrosone et al. [52] reported that the use of antioxidant supplements such as vitamin A and carotenoids was associated with an increased risk of cancer recurrence in breast cancer patients both before and during chemotherapy. Although there were no significant differences regarding the type of treatment, such as chemotherapy, hormone therapy, and radiation therapy, according to the flavonoid intake, the effect of flavonoids might be attenuated by some chemotherapeutic agents in the present study.

## 5. Conclusions

The present study showed that intake of flavonoids was associated with reduced risk of cancer recurrence, particularly in obese breast cancer patients, suggesting that increased consumption of flavonoid-rich foods could help prevent cancer recurrence. Further clinical studies are needed to confirm whether supplementation with flavonoids reduces cancer recurrence.

## Figures and Tables

**Table 1:** Characteristics of breast cancer patients with and without cancer recurrence.^1^

Variables	With Recurrence (*n* = 66)		Without Recurrence (*n* = 506)		*p*-Value *^2^*
	BMI < 23 (*n* = 33)	BMI ≥ 23 (*n* = 33)	BMI < 23 (*n* = 252)	BMI ≥ 23 (*n* = 254)	
BMI (kg/m^2^)	21.1 ± 1.3 ^a3^	26.5 ± 2.3 ^c^	20.7 ± 1.5 ^a^	25.7 ± 2.4 ^b^	<0.001
Age (years)	49.0 ± 9.5 ^a^	51.5 ± 11.5 ^a^	50.3 ± 9.7 ^a^	53.8 ± 10.9 ^b^	<0.001
WC (cm)	70.5 ± 3.5 ^a^	81.1 ± 6.6 ^c^	70.1 ± 5.7 ^a^	77.9 ± 6.5 ^b^	<0.001
FH, *n* (%)	4 (14.8)	4 (18.2)	31 (13.9)	32 (13.8)	0.952
Drinking, *n* (%)					0.002
Never	16 (48.5)	25 (75.8)	156 (61.9)	155 (61.3)	
Former	8 (24.2)	3 (9.1)	77 (30.6)	77 (30.4)	
Current	9 (27.3)	5 (15.2)	19 (7.5)	21 (8.3)	
Smoking, *n* (%)					0.934
Never	31 (93.9)	32 (97.0)	235 (93.3)	237 (93.7)	
Former	2 (6.1)	1 (3.0)	13 (5.2)	11 (4.3)	
Current	0 (0.0)	0 (0.0)	4 (1.6)	5 (2.0)	
Tumor, n (%)					0.003
<2 cm	19 (57.6)	9 (27.3)	155 (61.5)	139 (54.9)	
≥2 cm	14 (42.4)	24 (72.7)	97 (38.5)	114 (45.1)	
Lymph node metastasis, *n* (%)					0.012
Absence	24 (72.7)	19 (57.6)	197 (78.2)	171 (67.3)	
Presence	9 (27.3)	14 (42.4)	55 (21.8)	83 (32.7)	
AJCC, *n* (%)					0.075
Stages 0–2	28 (84.4)	25 (75.8)	226 (90.8)	220 (87.3)	
Stage 3	5 (15.2)	8 (24.2)	23 (9.2)	32 (12.7)	
Treatment, *n* (%)					
Chemotherapy	22 (66.7)	22 (68.8)	124 (56.6)	126 (55.0)	0.331
Hormonal therapy	24 (72.7)	21 (65.6)	169 (77.2)	165 (72.1)	0.427
Radiotherapy	11 (33.3)	15 (46.9)	121 (55.3)	128 (55.9)	0.080
Histologic grade, *n* (%)					0.405
Grade 1	8 (24.2)	4 (12.5)	62 (26.1)	67 (27.3)	
Grade 2	11 (33.3)	15 (46.9)	103 (43.3)	108 (44.1)	
Grade 3	14 (42.4)	13 (40.6)	73 (30.7)	70 (28.6)	
ER-negative, *n* (%)	11 (33.3)	14 (42.4)	71 (28.4)	77 (30.4)	0.413
PR-negative, *n* (%)	12 (36.4)	17 (51.5)	78 (31.2)	86 (34.0)	0.140
HER2, *n* (%)					0.705
Not amplified	23 (69.7)	21 (63.6)	183 (73.2)	181 (71.5)	
Amplification	10 (30.3)	12 (36.4)	67 (26.8)	72 (28.5)	
Subtype, *n* (%)					0.901
HR+/HER2−	20 (60.6)	15 (45.5)	151 (60.4)	145 (57.3)	
HR+/HER2+	6 (18.2)	8 (24.2)	42 (16.8)	41 (16.2)	
HR+/HER2+	4 (12.1)	4 (12.1)	25 (10.0)	31 (12.3)	
HR−/HER2−	3 (9.1)	6 (18.1)	32 (12.8)	36 (14.2)	

BMI, body mass index; WC, Waist circumference; FH, Family history of breast cancer; Drinking status, smoking status, AJCC stage, American Joint Committee on Cancer; ER expression, estrogen receptor; PR expression, progesterone receptor; HER2, human epidermal growth factor receptor 2; HR, hormone receptor; ^1^ values are presented as mean ± standard deviation or number of patients (percentage distribution) accordingly; ^2^ *p*-values were analyzed using the Kruskal–Wallis test for continuous variables and the chi-square test for categorical variables; ^3^ values indicated using different superscript letters in the same row are significantly different at *p* < 0.05 according to ANOVA with the LSD post hoc test.

**Table 2:** Consumption of flavonoids in patients with and without cancer recurrence.^1^

	With Recurrence (*n* = 66)		Without Recurrence (*n* = 506)		*p*-Value *^2^*	*p*-Value for Flavonoids × BMI Interaction ^4^
	BMI < 23 (*n* = 33)	BMI ≥ 23 (*n* = 33)	BMI < 23 (*n* = 252)	BMI ≥ 23 (*n* = 254)		
Flavonoids (mg/d)	60.84 ± 34.03 ^b3^	30.62 ± 20.99 ^a^	63.23 ± 45.81 ^b^	57.11 ± 41.08 ^b^	0.001	0.002
Flavonols (mg/d)	37.97 ± 29.21 ^b^	18.32 ± 14.07 ^a^	40.73 ± 41.12 ^b^	38.76 ± 36.09 ^b^	0.007	0.041
Quercetin (mg/d)	26.78 ± 17.99 ^ab^	11.67 ± 13.43 ^a^	29.36 ± 27.83 ^b^	27.15 ± 22.68 ^b^	0.018	0.008
Kaempferol (mg/d)	8.09 ± 6.83 ^ab^	4.17 ± 3.35 ^a^	7.85 ± 7.17 ^b^	8.09 ± 9.47 ^b^	0.011	0.030
Isorhamnetin (mg/d)	3.10 ± 3.50	2.48 ± 2.22	3.53 ± 4.11	3.52 ± 3.54	0.691	0.584
Flavone (mg/d)	22.24 ± 12.51 ^b^	12.29 ± 11.33 ^a^	22.51 ± 15.65 ^b^	18.35 ± 15.38 ^b^	0.007	0.003
Apigenin (mg/d)	9.09 ± 4.50 ^ab^	4.61 ± 3.71 ^a^	9.08 ± 7.89 ^b^	9.44 ± 11.55 ^b^	0.004	0.005
Luteolin (mg/d)	8.91 ± 6.43 ^ab^	7.68 ± 8.40 ^a^	13.42 ± 9.69 ^b^	13.15 ± 9.02 ^b^	0.010	0.020
Flavonoid-rich foods (g/d)	101.33 ± 63.99 ^b^	61.77 ± 53.44 ^a^	109.76 ± 79.90 ^b^	111.59 ± 80.60 ^b^	0.005	<0.001
Flavonol-rich foods (g/d)	65.63 ± 44.03 ^ab^	41.04 ± 42.30 ^a^	64.93 ± 56.89 ^b^	72.11 ± 63.64 ^b^	0.025	0.009
Flavone-rich foods (g/d)	35.70 ± 31.48 ^ab^	20.73 ± 24.23 ^a^	44.83 ± 42.55 ^b^	39.48 ± 37.77 ^b^	0.045	0.030

^1^ Values are presented as mean ± standard deviation; ^2^ *p*-values were determined by ranked analysis of variance for dietary intake of flavonoids or flavonoid-rich foods after adjusting for age, waist circumference, alcohol drinking, tumor size, lymph node metastasis, histologic grade, and energy intake; ^3^ values with different superscript letters in the same row are significantly different at *p* < 0.05, according to the ranked analysis of variance with the LSD post hoc test; ^4^ *p*-value for the interaction between flavonoids and BMI was determined using a two-way ANOVA; BMI, body mass index.

**Table 3:** Association between dietary intake of flavonoids and cancer recurrence in overweight and obese patients.

	Tertiles of Flavonoid Intake			*p* for Trend ^1^	Continuous
	T1	T2	T3		
Flavonoids					
Cutoff (range), 10 mg/d	2.14 (≤3.15)	4.27 (3.15 < to ≤ 5.78)	8.51 (>5.78)		
No. with/without recurrence	19/76	8/88	6/90		
Crude HR (95% CI)	1.0	0.426 (0.18–0.97)	0.299 (0.11–0.74)	0.011	0.967 (0.95−0.98)
Adjusted HR (95% CI) ^2^	1.0	0.208 (0.08–0.52)	0.249 (0.09–0.64)	0.008	0.959 (0.93−0.98)
Flavonols					
Cutoff (range), 10 mg/d	0.91 (≤1.45)	2.45 (1.45 < to ≤ 3.56)	6.80 (>3.56)		
No. with/without recurrence	18/78	9/86	6/90		
Crude HR (95% CI)	1.0	0.479 (0.21–1.06)	0.325 (0.12–0.82)	0.026	0.945 (0.91−0.97)
Adjusted HR (95% CI) ^2^	1.0	0.299 (0.12–0.70)	0.274 (0.99–0.76)	0.024	0.943 (0.90−0.97)
Quercetin					
Cutoff (range), 10 mg/d	0.36 (≤0.64)	1.06 (0.64 < to ≤ 2.52)	5.00 (>2.52)		
No. with/without recurrence	19/77	8/87	6/90		
Crude HR (95% CI)	1.0	0.441 (0.19–1.00)	0.325 (0.13–0.81)	0.041	0.972 (0.95−0.99)
Adjusted HR (95% CI) ^2^	1.0	0.353 (0.14–0.84)	0.301 (0.11–0.82)	0.047	0.966 (0.93−0.99)
Kaempferol					
Cutoff (range), 10 mg/d	0.21 (≤0.33)	0.46 (0.33 < to ≤ 0.75)	1.25 (>0.75)		
No. with/without recurrence	19/76	8/89	6/89		
Crude HR (95% CI)	1.0	0.389 (0.17–0.88)	0.275 (0.11–0.69)	0.013	0.865 (0.77−0.96)
Adjusted HR (95% CI) ^2^	1.0	0.427 (0.17–1.04)	0.268 (0.10–0.69)	0.014	0.868 (0.78−0.96)
Isorhamnetin					
Cutoff (range), 10 mg/d	0.09 (≤0.16)	0.23 (0.16 < to ≤ 0.34)	0.53 (>0.34)		
No. with/without recurrence	16/79	9/87	8/88		
Crude HR (95% CI)	1.0	0.533 (0.23–1.20)	0.523 (0.22–1.22)	0.167	0.899 (0.77−1.04)
Adjusted HR (95% CI) ^2^	1.0	0.523 (0.21–1.25)	0.428 (0.17–1.03)	0.080	0.901 (0.77−1.04)
Flavones					
Cutoff (range), 10 mg/d	0.59 (≤1.01)	1.50 (1.01 < to ≤ 2.04)	2.62 (>2.04)		
No. with/without recurrence	19/76	8/88	6/90		
Crude HR (95% CI)	1.0	0.391 (0.17–0.89)	0.290 (0.11–0.72)	0.005	0.946 (0.91−0.98)
Adjusted HR (95% CI) ^2^	1.0	0.403 (0.16–0.98)	0.337 (0.13–0.86)	0.015	0.948 (0.91−0.98)
Apigenin					
Cutoff (range), 10 mg/d	0.23 (≤0.45)	0.67 (0.45 < to ≤ 0.89)	1.30 (>0.89)		
No. with/without recurrence	20/76	7/88	6/90		
Crude HR (95% CI)	1.0	0.332 (0.14–0.78)	0.266 (0.10–0.66)	0.003	0.838 (0.73−0.95)
Adjusted HR (95% CI) ^2^	1.0	0.464 (0.18–1.14)	0.317 (0.12–0.81)	0.015	0.835 (0.71−0.95)
Luteolin					
Cutoff (range), 10 mg/d	0.13 (≤0.45)	0.86 (0.45 < to ≤ 1.30)	1.35 (>1.30)		
No. with/without recurrence	17/79	10/85	6/90		
Crude HR (95% CI)	1.0	0.588 (0.26–1.28)	0.315 (0.12–0.80)	0.011	0.974 (0.95−0.99)
Adjusted HR (95% CI) ^2^	1.0	0.409 (0.17–0.93)	0.315 (0.11–0.83)	0.007	0.971 (0.94−0.99)

^1^ Estimates of *p*-values for linear trends were based on linear scores derived from the medians of the tertiles of flavonoid intake in overweight and obese patients; ^2^ adjusted hazard ratio (HR) and 95% confidence interval (CI) were analyzed using Cox proportional hazards regression analysis after adjusting for age, waist circumference, alcohol drinking, tumor size, lymph node metastasis, histological grade, and energy intake.

**Table 4:** Association between dietary intake of flavonoid-rich foods and cancer recurrence in overweight and obese patients.

	Tertiles of Flavonoid-Rich Food Intake			*p* for Trend ^1^	Continuous
	T1	T2	T3		
Flavonoid-rich food					
Cutoff (range), 10 g/d	3.52 (≤5.99)	9.25 (5.99 < to ≤ 12.54)	16.75 (>12.54)		
No. with/without recurrence	19/75	8/88	6/91		
Crude HR (95% CI)	1.0	0.356 (0.15–0.81)	0.285 (0.11–0.71)	0.005	0.986 (0.97−0.99)
Adjusted HR (95% CI)^2^	1.0	0.294 (0.11–0.72)	0.244 (0.09–0.66)	0.006	0.985 (0.97−0.99)
Flavonol-rich food					
Cutoff (range), 10 g/d	0.85 (≤3.74)	5.95 (3.74 < to ≤ 8.24)	11.95 (> 8.24)		
No. with/without recurrence	17/75	9/87	7/92		
Crude HR (95% CI)	1.0	0.416 (0.18–0.93)	0.339 (0.14–0.81)	0.012	0.979 (0.96−0.99)
Adjusted HR (95% CI)^2^	1.0	0.384 (0.16–0.88)	0.321 (0.14–0.81)	0.014	0.979 (0.95−0.99)
Flavone-rich food					
Cutoff (range), 10 g/d	0.00 (≤1.79)	2.80 (1.79 < to ≤ 4.49)	6.80 (> 4.49)		
No. with/without recurrence	19/72	9/91	5/91		
Crude HR (95% CI)	1.0	0.418 (0.18–0.92)	0.260 (0.09–0.69)	0.005	0.986 (0.97−0.99)
Adjusted HR (95% CI)^2^	1.0	0.421 (0.18–0.97)	0.284 (0.10–0.79)	0.012	0.985 (0.97−0.99)

^1^ Estimates of *p*-values for linear trends were based on linear scores derived from the medians of tertiles of flavonoid-rich food intake in overweight and obese patients; ^2^ adjusted hazard ratios (HRs) and 95% confidence intervals (CIs) were analyzed using Cox proportional hazards regression analysis after adjusting for age, waist circumference, alcohol drinking, tumor size, lymph node metastasis, histological grade, and energy intake.

## Data Availability

The dataset for this study is available from the corresponding author on reasonable request.

## Supplementary Materials

The following are available online at www.mdpi.com/article/10.3390/nu13093049/s1, Supplement file 1: Figure S1. Survival curves according to tertiles of flavonoids or flavonoid-rich foods. Cumulative overall survival according to tertiles of flavonoids or flavonoid-rich foods in overweight and obese patients with breast cancer, Figure S2. Survival curves according to tertiles of flavonoids or flavonoid-rich foods. Cumulative disease-free survival and overall survival according to tertiles of flavonoids or flavonoid-rich foods in all patients with breast cancer, Supplement file 2: Table S1: Biochemical parameters in patients with and without cancer recurrence, Table S2: Association between dietary intake of flavonoids and cancer recurrence risk in all breast cancer patients, Table S3: Association between dietary intake of flavonoid-rich foods and cancer recurrence risk in breast cancer patients.
