# Supplementary material for: Association between Dietary Intake of Flavonoids and Cancer Recurrence among Breast Cancer Survivors
Source: Nutrients. 2021 Aug 30;13(9):3049. doi: 10.3390/nu13093049 (PMC8469315; doi:10.3390/nu13093049)
Supplement: Supplementary file 1 [file nutrients-13-03049-s001.zip › Supplemental Tables.pdf]

Supplemental Table 1. Biochemical parameters in patients with and without cancer recurrence[1]

	With recurrence (n = 66)		Without recurrence (n = 506)		p-value[2]
	BMI < 23 (n = 33)	BMI ≥ 23 (n = 33)	BMI < 23 (n = 252)	BMI ≥ 23 (n = 254)	
TP (g/L)	74.31 ± 0.36	72.83 ± 0.61	73.32 ± 0.52	72.91 ± 0.48	0.327
ALB (g/L)	44.52 ± 0.25	42.81 ± 0.68	43.83 ± 0.35	43.74 ± 0.50	0.451
ALP (μkat/L)	7.04 ± 1.86	6.88 ± 7.26	7.20 ± 3.76	7.94 ± 2.70	0.086
AST (μkat/L)	2.31 ± 0.76	6.11 ± 1.18	2.29 ± 0.90	2.63 ± 1.38	0.134
ALT (μkat/L)	2.13 ± 0.94	3.09 ± 5.04	2.11 ± 1.27	2.33 ± 1.42	0.855
WBC (10^9/L)	5.40 ± 1.56	7.46 ± 4.51	5.69 ± 1.89	5.96 ± 2.11	0.518
Hemoglobin (g/L)	122.71 ± 13.62	121.92 ± 16.43	124.94 ± 12.52	128.15 ± 64.71	0.740
Hematocrit (%)	36.77 ± 3.91	36.76 ± 4.61	37.48 ± 3.43	37.36 ± 4.61	0.818
TG (mmol/L)	1.37 ± 0.64	1.80 ± 0.10	1.48 ± 0.91	1.76 ± 0.90	0.120
HDL–C (mmol/L)	5.25 ± 1.42	4.50 ± 1.07	5.54 ± 1.28	5.10 ± 1.17	0.333
LDL–C (mmol/L)	9.16 ± 2.37	12.28 ± 4.76	10.35 ± 2.80	11.14 ± 3.35	0.611
Cholesterol (mmol/L)	19.13 ± 3.33	18.50 ± 6.51	18.87 ± 4.33	19.28 ± 3.95	0.783
CA15–3 (U/mL)	10.03 ± 4.82	18.71 ± 43.07	9.68 ± 5.28	10.01 ± 6.77	0.622

TP, total protein; ALB, albumin; ALP, alkaline phosphatase; AST, aspartate aminotransferase; ALT, alanine aminotransferase; TG, triglycerides; HDL–C, high–density lipoprotein cholesterol; LDL–C, low–density lipoprotein cholesterol; WBC, white blood cell; CA15–3, cancer antigen 15–3; [1]values are mean ± SD; [2] p–values are determined by ranked–ANOVA after adjusting for age, waist circumference, alcohol drinking, tumor size, lymph node metastasis, histologic grade, and energy intake. BMI, body mass index.

Supplemental Table 2. Association between dietary intake of flavonoids and the risk of cancer recurrence in all breast cancer patients

	Tertiles of flavonoid intake			*p* for trend[1]	Continuous
	T1	T2	T3		
Flavonoids					
Cut off (range), 10 mg/d	2.36 (≤ 3.30)	4.57 (3.30 < to ≤ 6.25)	9.59 (> 6.25)		
No. of with/without recurrence	29/161	18/173	19/172		
Crude HR (95% CI)	1.0	0.634 (0.35–1.14)	0.622 (0.34–1.11)	0.154	0.990 (0.98-1.04)
Adjusted HR (95% CI)[2]	1.0	0.547 (0.29–1.02)	0.727 (0.40–1.31)	0.421	0.992 (0.98-1.05)
Flavonols					
Cut off (range), 10 mg/d	1.00 (≤ 1.55)	2.45 (1.55 < to ≤ 3.62)	7.13 (> 3.62)		
No. of with/without recurrence	28/162	20/171	18/173		
Crude HR (95% CI)	1.0	0.663 (0.37–1.17)	0.603 (0.33–1.09)	0.151	0.989 (0.97-1.01)
Adjusted HR (95% CI)[2]	1.0	0.586 (0.32–1.05)	0.621 (0.33–1.15)	0.242	0.992 (0.98-1.02)
Quercetin					
Cut off (range), 10 mg/d	0.41 (≤ 0.74)	1.12 (0.74 < to ≤ 2.48)	5.55 (> 2.48)		
No. of with/without recurrence	25/165	25/166	16/175		
Crude HR (95% CI)	1.0	1.079 (0.62–1.87)	0.647 (0.34–1.21)	0.110	0.989 (0.97-1.03)
Adjusted HR (95% CI)[2]	1.0	1.057 (0.60–1.85)	0.693 (0.35–1.33)	0.207	0.992 (0.98-1.02)
Kaempferol					
Cut off (range), 10 mg/d	0.22 (≤ 0.34)	0.52 (0.34 < to ≤ 0.82)	1.25 (> 0.82)		
No. of with/without recurrence	28/162	20/171	18/173		
Crude HR (95% CI)	1.0	0.731 (0.41–1.29)	0.623 (0.34–1.12)	0.143	0.961 (0.91-1.02)
Adjusted HR (95% CI)[2]	1.0	0.815 (0.45–1.47)	0.694 (0.37–1.27)	0.263	0.967 (0.92-1.06)
Isorhamnetin					
Cut off (range), 10 mg/d	0.08 (≤ 0.15)	0.23 (0.15 < to ≤ 0.33)	0.54 (> 0.33)		
No. of with/without recurrence	29/161	21/170	16/175		
Crude HR (95% CI)	1.0	0.709 (0.40–1.24)	0.581 (0.30–1.07)	0.093	0.946 (0.86-1.03)
Adjusted HR (95% CI)[2]	1.0	0.710 (0.39–1.26)	0.567 (0.30–1.07)	0.093	0.950 (0.87-1.03)
Flavones					
Cut off (range), 10 mg/d	0.74 (≤ 1.24)	1.64 (1.24 < to ≤ 2.13)	3.18 (> 2.13)		
No. of with/without recurrence	29/160	17/175	20/171		
Crude HR (95% CI)	1.0	0.582 (0.32–1.05)	0.645 (0.36–1.14)	0.170	0.983 (0.96-1.02)
Adjusted HR (95% CI)[2]	1.0	0.623 (0.33–1.14)	0.738 (0.41–1.31)	0.367	0.987 (0.96-1.05)
Apigenin					
Cut off (range), 10 mg/d	0.26 (≤ 0.51)	0.66 (0.51 < to ≤ 0.91)	1.39 (> 0.91)		
No. of with/without recurrence	27/163	18/174	21/169		
Crude HR (95% CI)	1.0	0.694 (0.38–1.26)	0.779 (0.44–1.37)	0.466	0.949 (0.89-1.03)
Adjusted HR (95% CI)[2]	1.0	0.815 (0.44–1.50)	0.874 (0.48–1.56)	0.701	0.948 (0.89-1.02)
Luteolin					
Cut off (range), 10 mg/d	0.22 (≤ 0.60)	0.93 (0.60 < to ≤ 1.34)	1.60 (> 1.34)		
No. of with/without recurrence	27/163	19/154	20/189		
Crude HR (95% CI)	1.0	0.785 (0.43–1.41)	0.607 (0.34–1.08)	0.090	0.987 (0.95-1.01)
Adjusted HR (95% CI)[2]	1.0	0.650 (0.35–1.19)	0.647 (0.35–1.16)	0.139	0.994 (0.96-1.02)

[1]Estimates of *p* for trend values for linear trends are based on linear scores derived from the medians of tertiles of flavonoid intake among all patients; [2]adjusted hazard ratio (HR) and 95% confidence interval (CI) are analyzed via Cox proportional hazards regression analysis after adjusted for age, waist circumference, alcohol drinking, tumor size, lymph node metastasis, histologic grade, and energy intake.

Supplemental Table 3. Association between dietary intake of flavonoid–rich foods and the risk of cancer recurrence in all breast cancer patients

	Tertiles of flavonoid-rich food intake			*p* for trend[1]	Continuous
	T1	T2	T3		
Flavonoid–rich food					
Cut off (range), 10 g/d	3.75 (≤ 6.61)	9.50 (6.61 < to ≤ 12.57)	17.10 (> 12.57)		
No. of with/without recurrence	29/161	18/173	19/172		
Crude HR (95% CI)	1.0	0.560 (0.31–1.01)	0.627 (0.35–1.11)	0.122	0.996 (0.99-1.05)
Adjusted HR (95% CI)2	1.0	0.593 (0.32–1.09)	0.719 (0.39–1.30)	0.292	0.998 (0.99-1.06)
Flavonol–rich food					
Cut off (range), 10 g/d	0.97 (≤ 3.50)	5.51 (3.50 < to ≤ 8.15)	11.50 (> 8.15)		
No. of with/without recurrence	26/158	21/176	19/172		
Crude HR (95% CI)	1.0	0.595 (0.33–1.05)	0.640 (0.35–1.15)	0.159	0.992 (0.98-1.02)
Adjusted HR (95% CI)2	1.0	0.613 (0.34–1.09)	0.668 (0.36–1.22)	0.213	0.994 (0.98-1.03)
Flavone–rich food					
Cut off (range), 10 g/d	0.00 (≤ 2.30)	3.30 (2.30 < to ≤ 4.54)	7.20 (> 4.54)		
No. of with/without recurrence	28/153	22/178	16/175		
Crude HR (95% CI)	1.0	0.720 (0.41–1.25)	0.555 (0.30–1.02)	0.058	0.998 (0.98-1.02)
Adjusted HR (95% CI)2	1.0	0.757 (0.42–1.36)	0.639 (0.33–1.20)	0.163	0.997 (0.98-1.01)

[1]Estimates of *p* for trend values for linear trends are based on linear scores derived from the medians of tertiles of flavonoid–rich food intake among all patients; [2]adjusted hazard ratio (HR) and 95% confidence interval (CI) are analyzed via Cox proportional hazards regression analysis after adjusted for age, waist circumference, alcohol drinking, tumor size, lymph node metastasis, histologic grade, and energy intake.
