# Supplementary figures and images for: Association between Dietary Intake of Flavonoids and Cancer Recurrence among Breast Cancer Survivors
Source: Nutrients. 2021 Aug 30;13(9):3049. doi: 10.3390/nu13093049 (PMC8469315; doi:10.3390/nu13093049)

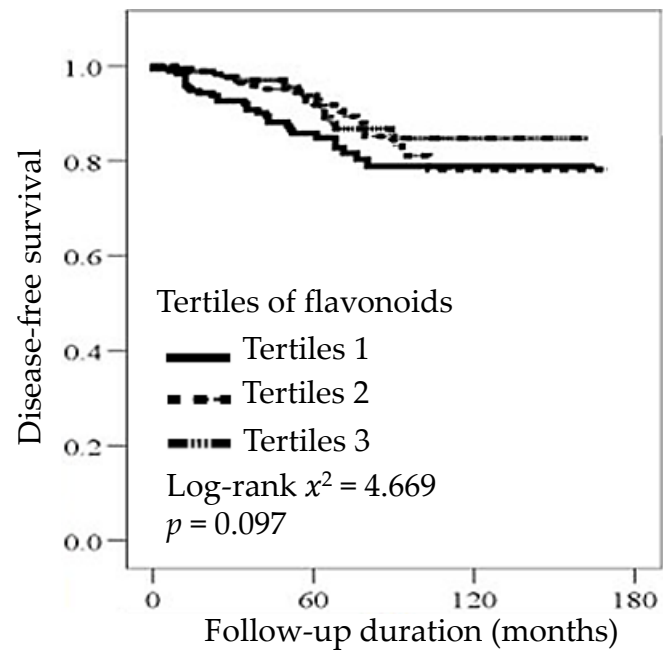

(a)

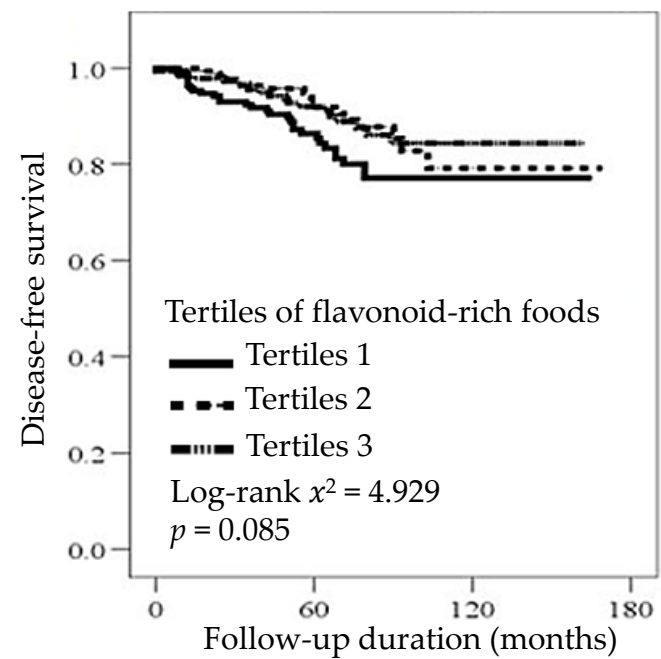

(b)

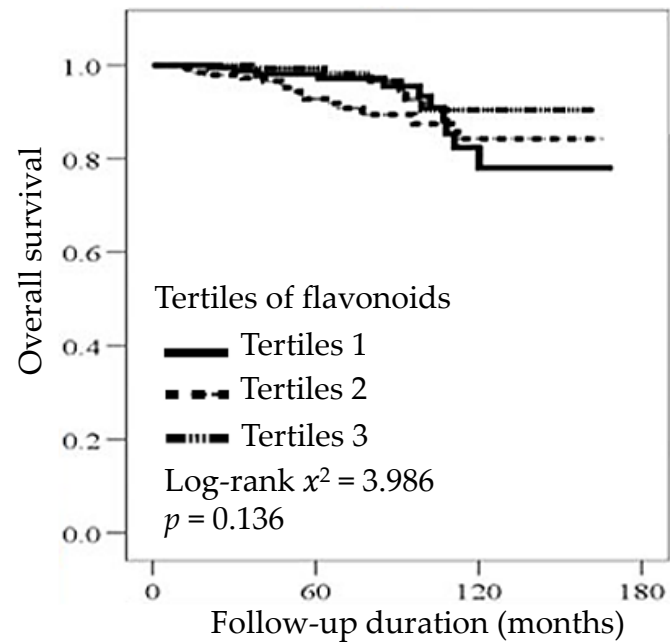

(c)

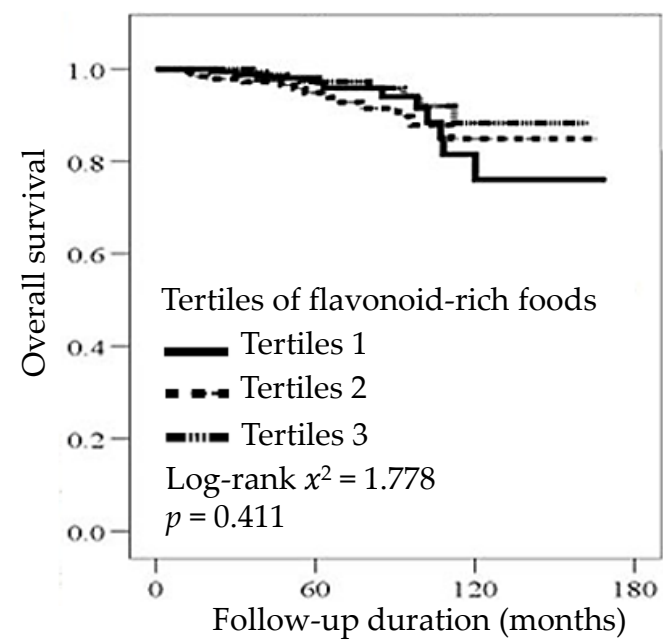

(d)

Supplement: Supplementary file 1 [file nutrients-13-03049-s001.zip › Fig S1.pdf]

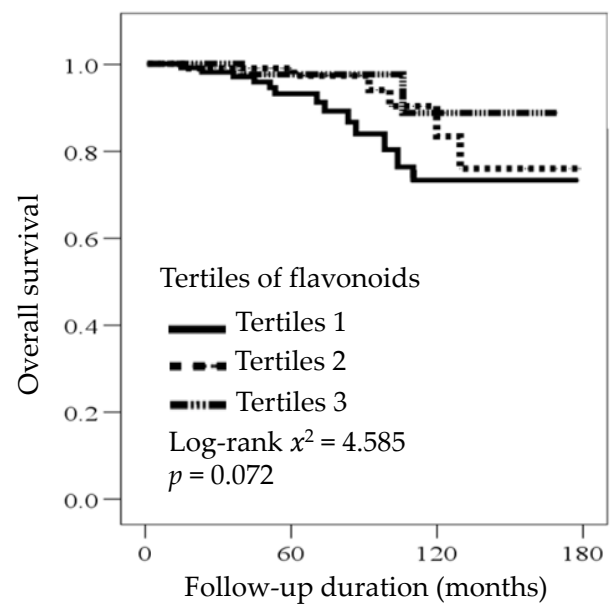

(a)

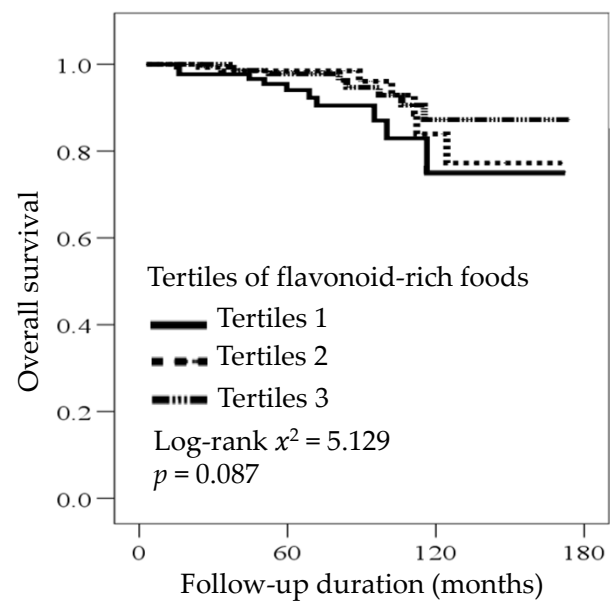

(b)

Supplement: Supplementary file 1 [file nutrients-13-03049-s001.zip › Fig S2.pdf]
